# Supplementary material for: Improving CNV Detection Performance in Microarray Data Using a Machine Learning-Based Approach
Source: Diagnostics (Basel). 2023 Dec 29;14(1):84. doi: 10.3390/diagnostics14010084 (PMC10871075; doi:10.3390/diagnostics14010084)
Supplement: Supplementary file 1 [file diagnostics-14-00084-s001.zip › Supplementary Figure S1. Examples of next-generation sequencing validation of CNV-related.pdf]

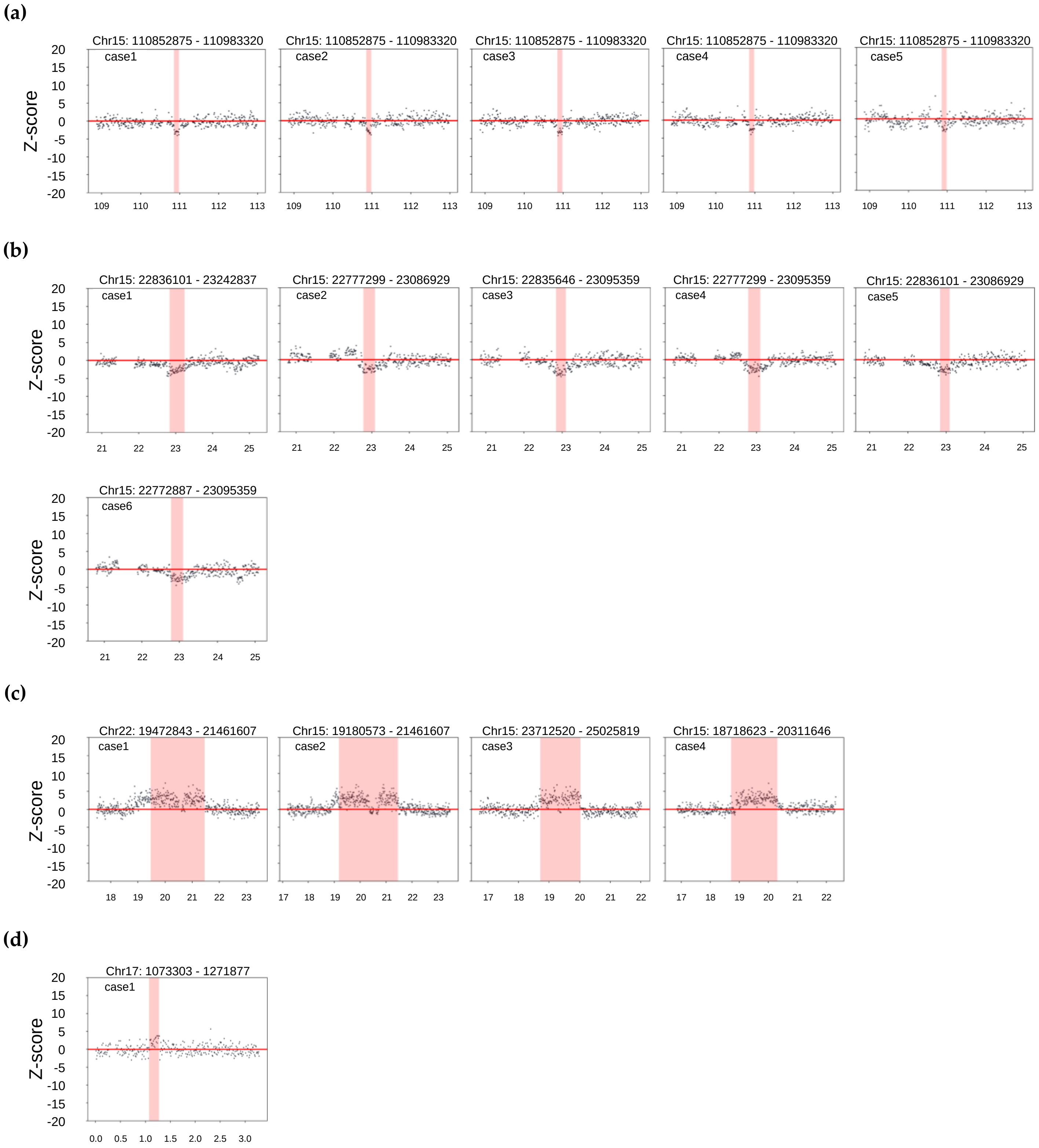
(a)
Chr15: 110852875 - 110983320
case1
case2
case3
case4
case5
Z-score
(b)
Chr15: 22836101 - 23242837
case1
Chr15: 22777299 - 23086929
case2
Chr15: 22835646 - 23095359
case3
Chr15: 22777299 - 23095359
case4
Chr15: 22836101 - 23086929
case5
Chr15: 22772887 - 23095359
case6
(c)
Chr22: 19472843 - 21461607
case1
Chr15: 19180573 - 21461607
case2
Chr15: 23712520 - 25025819
case3
Chr15: 18718623 - 20311646
case4
(d)
Chr17: 1073303 - 1271877
case1

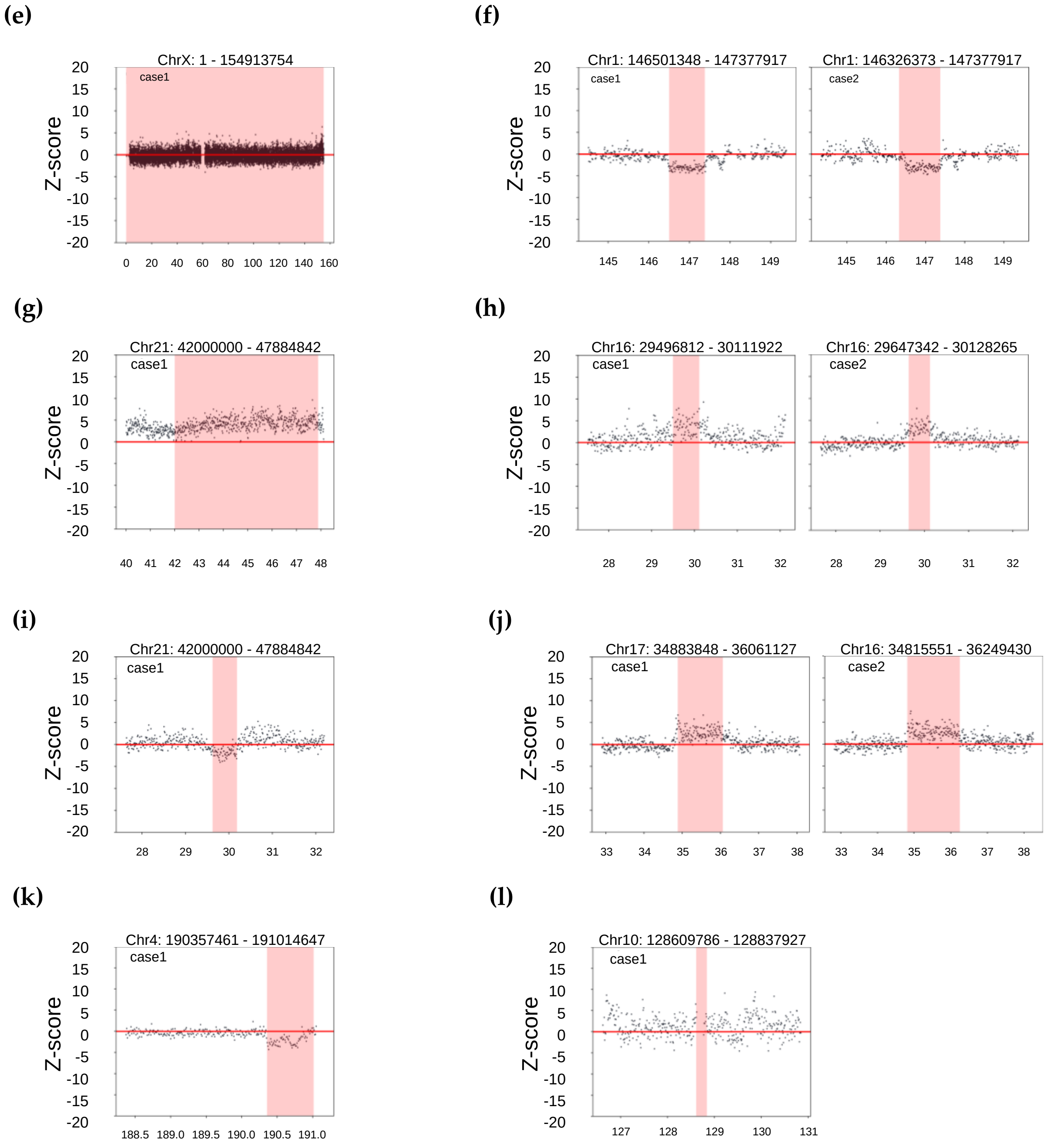

Figure S1. Examples of next generation sequencing validation of CNV-related chromosomal disorder. Of a total of 342 detected CNV-associated chromosomal disorders, 37 cases were validated by NGS. Among them, the NGS results for 28 cases were shown. Y-axis indicates Z-score, and X-axis represents positions (Mb): **(a)** Joubert syndrome 4; **(b)** 15q11.2 deletion syndrome; **(c)** 22q11.2 duplication syndrome; **(d)** 17p13.3; telomeric duplication syndrome; **(e)** Klinefelter syndrome; **(f)** 1q21.1 microdeletion syndrome; **(g)** Down syndrome; **(h)** 16p11.2 duplication syndrome; **(i)** 16p11.2 microdeletion syndrome (593 kb); **(j)** 17q12 duplicate on syndrome; **(k)** Facioscapulohumeral muscular dystrophy; **(l)** 10q26 deletion syndrome.
